# Construction of cell-plastics as neo-plastics consisted of cell-layer provided green alga *Chlamydomonas reinhardtii* covered by two-dimensional polymer

**DOI:** 10.1186/s13568-020-01046-y

**Published:** 2020-06-10

**Authors:** Akihito Nakanishi, Kohei Iritani, Yuri Sakihama, Nanami Ozawa, Ayano Mochizuki, Marina Watanabe

**Affiliations:** 1 Graduate School of Bionics, Tokyo University of Technology, 1404-1 Katakuramachi, Hachioji, Tokyo, 192-0982 Japan; 2 School of Bioscience and Biotechnology, Tokyo University of Technology, 1404-1 Katakuramachi, Hachioji, Tokyo, 192-0982 Japan; 3 Department of Applied Chemistry, School of Engineering, Tokyo University of Technology, Hachioji, Japan; 4 Tokyo University of Technology, Hachioji, Japan

**Keywords:** *Chlamydomonas reinhardtii*, Two-dimensional polymer, Stacking structure, Cell-plastics

## Abstract

Green alga *Chlamydomonas reinhardtii* has gained interest as a sustainable resource because it can be easily grown using CO_2_ as a carbon source owing to its high CO_2_ assimilating activity. Although the robustness of the cell wall of *C. reinhardtii* makes it difficult to extract its intracellular products, such property is beneficial when using the cell as an ingredient to fabricate “cell-plastic” in this study. The cell layer, which is a component of the cell-plastic, was prepared with an intercellular filler to connect each cell because *C. reinhardtii* is a single-cell strain. The cell layers were then repeatedly piled to increase the strength of the cell-plastic. To avoid slippage between the cell layers, they were covered with a small amount of a two-dimensional polymer to maintain the flat surface structure of the cell-plastic. Based on the evaluation, the cell-plastic has the potential to be a novel, sustainable plastic using ubiquitous green algal cells in nature. 
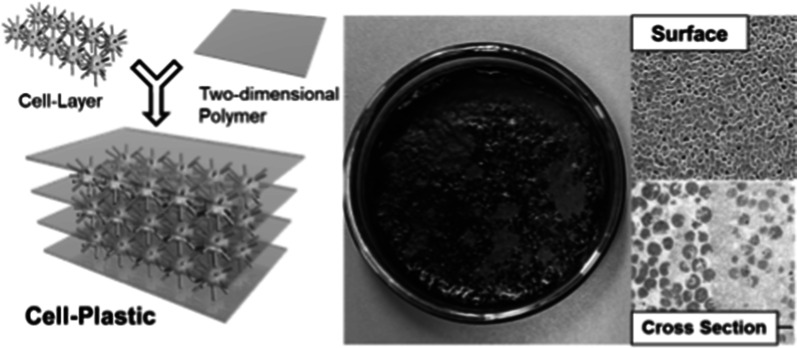

## Key Points

- Cell-plastic was fabricated with green algal cells with intercellular filler and two-dimensional polymer.
- Stacking structure was adopted for the cell-plastic because of improvement of its mechanical strength.
- Electron microscopy observations revealed nanostructures of cell-plastic.

## Introduction

Plastics, which consist of multiple materials polymerized with organic molecules, freely molded to produce fibers and films by heat and/or pressing. Polyethylene (PE) and polypropylene (PP) comprise the majority of plastics and are generally used as soft sheets and automobile parts (Garcés et al. 2000; Maddah 2016). PE and PP are produced in a cost-competitive way and possess the required durability and strength for daily use; thus, plastics have become essential materials in our society (Maddah 2016; Sohn et al. 2020). In fact, the total amount of plastics produced had reached 7.8 billion tons from 1950 to 2015, and half of that amount was produced in the past 13 years (Geyer et al. 2017). However, oil-based plastics have created many problems: oil consumption has been increasing, reaching 1.4 million barrels per day to develop and maintain the world society, while the oil deposit is limited (Shahbaz et al. 2014; Yao et al. 2020); the greenhouse effect has accelerated with increasing CO_2_ emission due to combustion of oil and molded plastics (Villanueva et al. 2019); 5 trillion undegraded plastic particles weighing over 250,000 tons are drifting at sea worldwide (Eriksen et al. 2014). Although many studies have been conducted to produce bioplastics derived from biomass products such as polylactic acid as a green plastic since the 1990s, the production of green plastics consumes a large amount of time and money; thus, only few green products have reached a practical level of production (Ammala et al. 2011; Iles et al. 2013). Therefore, new sustainable plastics using alternative sources to oil should be developed with a novel way of thinking.

Green alga *C. reinhardtii* is a monocellular microorganism producing biomass materials that assimilate CO_2_ as a carbon source (Salguero et al. 2018). Microalgae such as *C. reinhardtii* assimilate CO_2_ at a rate of 10–50 times greater than that of general terrestrial plants (Wang et al. 2008). Moreover, *C. reinhardtii* is a safe strain certified as *generally recognized as safe* (GRAS) (Ochoa-Méndez et al. 2016), indicating that it has the potential to be a biomass-producing strain. In fact, several studies have attempted to produce lipids and carotenoids from *C. reinhardtii* (Hang et al. 2020; Moon et al. 2013; Sun et al. 2018). The cell structure of *C. reinhardtii* is rigid; therefore, its cell wall generally needs to be broken sufficiently to extract products in the cell (Lee et al. 1996). The robustness of its cell wall is normally a disadvantage for extracting products, but it could be an advantage when it is used as an ingredient. Our study focused on the possibility of using the cell itself of *C. reinhardtii* as an ingredient for plastics. To use its cells as raw materials for plastics, an intercellular filler was considered to connect each cell because it is a monocellular microorganism. Thus, the cell layer was constructed with flocculated cells by using suitable intercellular fillers in this study. The cell layer would be a raw material for plastics if it could be processed using a mold. The material was called “cell-plastic” in this study.

**Fig. 1:**
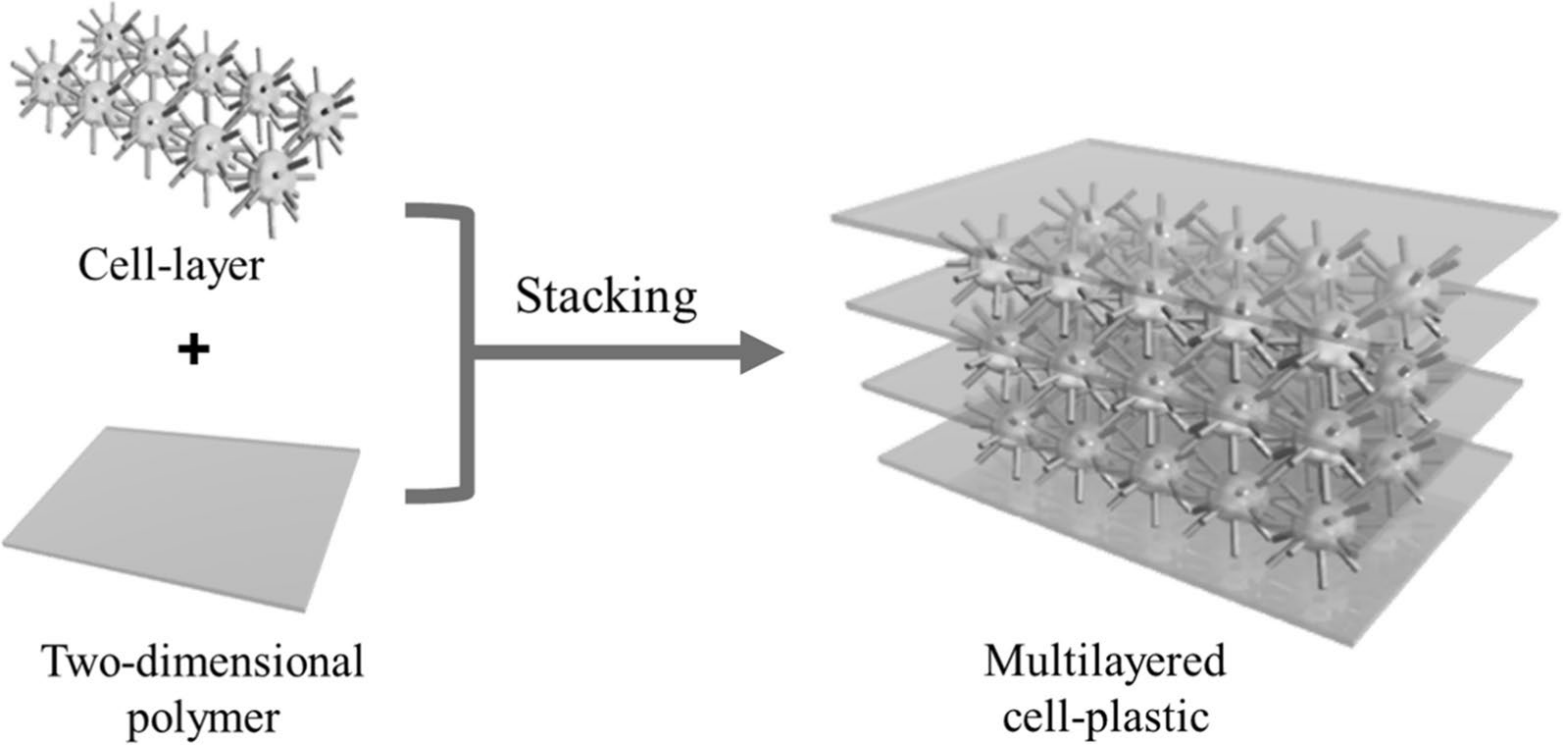
Schematic model of the concept of this work

**Fig. 2:**
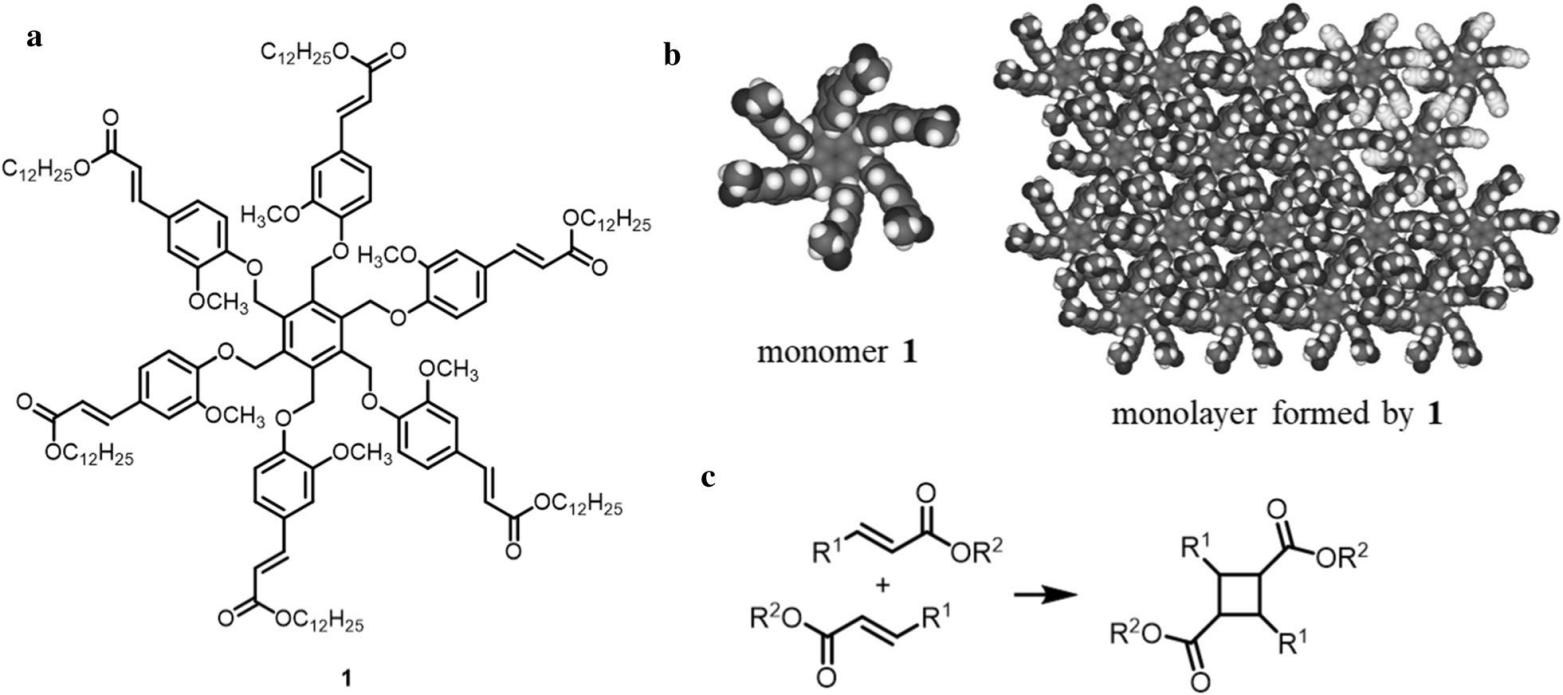
Chemical structure and molecular models **a** Chemical structure of monomer 1, **b** molecular models of 1 (left) and monolayer formed by 1 (right), and **c** chemical reaction of photocycloaddition of cinnamate derivative. In (**b**), C_12_ alkyl chains and some of the cinnamate units at an upper right part of the model of the monolayer are omitted for clarity reason

To develop flexible and high-strength cell-plastics, we proposed to stack the cell layers. As a supporting material for stacking the cell layers without slippage between them, we focused on a two-dimensional polymer (2DP), which is a covalently bonded molecular sheet consisting of repeating units with an internal 2D long-range order (Clair et al. 2019; Janica et al. 2018; Payamyar et al. 2016; Sakamoto et al. 2009) because a 2DP has flexibility owing to its thin film-like structure formed by organic molecules. Moreover, it maintains a plane structure even with using only a small amount to support the cell layers. As shown in Fig. 1, we propose an approach of fabricating cell-plastics by alternately stacking the cell layers and 2DPs. As reaction fields for the synthesis of 2DPs, the surface of substrates under ultrahigh vacuum (UHV) at high temperatures (Bieri et al. 2010; Lafferentz et al. 2012), solid/air or solid/liquid interfaces under atmospheric conditions (Guan et al. 2012; Yu et al. 2016), and air/water interface (Murray et al. 2015; Servalli et al. 2018) have been used. In this study, because the cells were used as ingredients for the cell-plastic, it is difficult to synthesize the 2DP under the condition of UHV at high temperatures. In addition, in synthetic methods using solid substrates, there is a problem of transferring the obtained 2DP from the substrates to the surface of the cell layer. Therefore, we adopted the air/water interface as a reaction field because the cells have resistance to water and air, and the 2DP can be transferred to the cell layers easily. To this end, we designed monomer **1** (Fig. 2a), which adopts a sixfold symmetric structure with methoxy units as a hydrophilic group, C_12_ alkyl chains as a hydrophobic group, and cinnamate groups as a reactive unit. We expected that monomer **1** would form a monolayer on the water surface, as illustrated in Fig. 2b. After formation of the monolayer, covalent bond formations between the cinnamate units of monomers would proceed by UV light irradiation (Fig. 2c) (Chung et al. 1991). As a pre-experiment, we found that long-term contact of the cell layers with water resulted in the collapse of the cell aggregation. Therefore, after construction of the cell layer on a glass Petri dish as a template to prevent deformation of the cell layer, monomer **1** was diffused on the water which was added to the cell layer. After 2DP was subsequently synthesized by photo-irradiation, another cell layer was constructed on the 2DP layer. By repeating this method, the stacking structure of the cell layer and 2DP to form the cell-plastic was achieved.

Our study attempted to produce cell-plastics as alternative materials to oil-based plastics using sustainable green algal cells. The fabrication of the cell-plastic was evaluated by analyzing the (a) culturing conditions to produce the cells of *C. reinhardtii* as the ingredient, (b) cell components, (c) Young’s modulus and tensile strength, and (d) surface and internal structures of the cell-plastic by transmission electron microscopy (TEM) and scanning electron microscopy (SEM) observations.

## Materials and methods

### Culturing conditions

*C. reinhardtii* strain C-9: NIES-2235 was cultured with a light intensity of 50 μmol photons·m^−2^·s^−1^ (white fluorescent lamps) at 23 °C in modified Bold (MB) 6 N medium, as described by a previous study (Ho et al. 2017). The cultivation was started with 1.0–2.0 × 10^6^ cells provided from a pre-culture in MB6N medium before nitrogen starvation. The aeration rate of 0.8% CO_2_ was 0.1 vvm, which was evaluated and controlled using a flow meter (Model RK1200 series, KOFLOC, Kyoto, Japan).

### Evaluation of broth

The cell numbers were evaluated with the value of OD_750_ via an appropriate calibration curve of cell numbers versus OD_750_. The pH was measured after centrifugation of the broth at 5000×*g* for 1 min. To measure the nitrate concentration, the permeate of the broth filtered with a 0.45 µm filter (Millex^Ⓡ^-LCR 13 mm, Millipore, Carrigtwohill, Ireland) was diluted 50-fold with distilled water. The absorbance of 220 nm was measured, and the nitrate concentration was evaluated with the value of Abs_220_ using an appropriate calibration curve. To measure the pH, the supernatant was placed on a digital pH meter (LAQUAtwin, Horiba, Kyoto, Japan).

### Determination of cell composition

The total lipid content was evaluated using gas chromatography with a flame ionization detector. Cells were collected by centrifugation at 5000×*g* for 1 min, washed with distilled water twice, and dried under vacuum. The dried cells were fractured with 0.5 mm glass beads using a vortex mixer at 23 °C. The total lipids were esterized and extracted using a fatty acid methylation kit (Nacalai Tesque, Kyoto, Japan). The fatty acid methyl esters were identified and quantified using a capillary gas chromatograph (GC-2025; Shimadzu, Kyoto, Japan) equipped with a DB-23 capillary column (60 m, 0.25 mm internal diameter, 0.15 μm film thickness; Agilent Technologies, Palo Alto, CA) with nitrogen as the carrier gas at a flow rate of 2.3 mL·min^−1^. The oven temperature was initially set at 50 °C for 1 min, then increased from 50 to 175 °C at a rate of 25 °C·min^−1^, then increased from 175 to 230 °C at a rate of 4 °C·min^−1^, and finally held at 230 °C for 5 min. The injector, ion source, and interface source temperatures were set at 230, 230, and 250 °C, respectively. Heptadecanoic acid (Sigma-Aldrich Co., St. Louis, MO) was used as an internal standard, and rapeseed oil (Merck KGaA, Darmstadt, Germany) was used as a quantitative standard. The total carbohydrate content was evaluated using the anthrone-sulfuric acid method as described by Ho et al. (2017). The total protein content was determined using a bicinchoninic acid protein assay kit (Takara Bio, Shiga, Japan).

### Synthesis of monomer

The synthesis of monomer **1** is detailed in the Additional file 1: (Figs. S2, S3 and S4).

### Molecular modeling

The molecular model of monomer **1** was built using Winmostar software. The structure of the monolayer formed by monomer **1** (in which a sixfold symmetric structure, i.e., a honeycomb-type structure, was adopted (Fig. 2b)) was built using ViewerLight software. The distances between molecules and the angle among molecules were set as approximately 1.4 nm and 60°, respectively. The unit cell area was calculated to be approximately 1.7 nm^2^. In the model, the distances between the cinnamate units in the adjacent molecules were estimated to be less than 0.4 nm, which indicates that the distances are short enough to proceed with photocycloaddition (Chung et al. 1991).

### Synthesis of 2DP using a Langmuir–Blodgett (LB) trough

A KSV NIMA large LB trough (Biolin Scientific, Stockholm, Sweden) equipped with a platinum Wilhelmy plate was used. As a subphase, Millipore water (18.2 MΩ·cm) obtained from ultrapure water production equipment (RFU424BB; ADVANTEC Co., Ltd., Tokyo, Japan) was used. For spreading of monomer **1**, a stock solution (2.2 × 10^−4^ M) of chloroform was prepared. The solution was spread dropwise at the air/water interface with the use of a glass microsyringe. The typical volumes applied were 200 μL. After spreading, we waited for 30 min to allow complete evaporation of the solvents. To visualize the films on the water surface by Brewster angle microscopy (BAM), a KSV MicroBAM (Biolin Scientific, Stockholm, Sweden) operating with a 658 nm laser was used. The monomer monolayer was polymerized by direct photo-irradiation at 356 nm for 2 h using a handy UV lamp (AS ONE Co., Ltd., Tokyo, Japan) with 8 W of nominal power.

### SEM observation of 2DP

A TEM copper grid with a mesh size of 1000 (G2780C; EM Japan Co., Ltd., Tokyo, Japan,) was placed on the photo-irradiated monolayer. A white piece of paper was then gently positioned on the grid to collect it. After drying at room temperature, the photo-irradiated monolayer on the TEM grid was observed using an SEM (JSM-6060LV; Japan Electron Optics Laboratory Co., Ltd., Tokyo, Japan).

### Construction of the monolayer cell-plastic with an intercellular filler

*C. reinhardtii* cells were harvested by centrifugation at 5000×*g* for 1 min at 23 °C, adjusted to collect almost 9.0 × 10^8^ cells. To wash the cells, the harvested cells were mixed with 20 mL of deionized water by vortexing. Then, they were centrifuged at 5000×*g* for 1 min at 23 °C, and the supernatant was discarded. The processes were repeated twice. The washed cells were loaded onto Petri dishes (diameter: 5.5 cm) and added with each intercellular filler in two combinations: (i) 270 µL of 99.5% (*w*/*w*) glycerol; (ii) 270 µL of 99.5% (*w*/*w*) glycerol and 1340 µL of 1.0% (*w*/*v*) bovine serum albumin (BSA). After pipetting the cells with each intercellular filler, the mixture was dried for over 12 h in an oven at 80 °C. Further drying was performed under vacuum at 80 °C for 1.5 h. On the dried cell layer, Millipore water (approximately 500 μL) was placed as a foundation for monomer diffusion; subsequently, 100 μL of a chloroform solution of monomer **1** (2.2 × 10^−5^ M) was added dropwise with the use of a glass microsyringe. After 5 min, UV light irradiation at 356 nm was performed for 2 h using a handy UV lamp (AS ONE Co., Ltd., Tokyo, Japan) with 8 W of nominal power.

### Formation of the multilayered cell-plastic

The multilayered cell-plastic was formed from six layers of monolayer cell-plastics. After fabricating the monolayer cell-plastics following the method described in “Construction of the monolayer cell-plastic with an intercellular filler,” the monolayer cell-plastics were repeatedly laid on the dried cell-plastics. The cells comprising the 1st to 6th cell-plastic layers were harvested in the broth at 70 h (1st layer), 118 h (2nd layer), 166 h (3rd layer), 169 h (4th layer), 241 h (5th layer), and 295 h (6th layer).

### Electron microscopy observations

Images of the multilayered cell-plastics were taken using the SEM (JSM-6060LV; Japan Electron Optics Laboratory Co., Ltd., Tokyo, Japan). Before scanning, the cell-plastics were coated with Au particles using an ion coater (IB-2; Eiko Engineering, Tokyo, Japan). Then, TEM images of the multilayered cell-plastics were taken after preparing processes as below. Fixation: the samples were fixed with 2% paraformaldehyde and 2% glutaraldehyde in 0.05 M carbohydrate buffer pH 7.4 at 4 °C over 8 h. After this fixation, the samples were washed 3 times with 0.05 M cacodylate buffer for 30 min each, and were postfixed with 2% osmium tetroxide in 0.05 M cacodylate buffer at 4 °C for 3 h. Dehydration: the samples were dehydrated in graded ethanol solutions (50%, 70%, 90%, 100%). The schedule was as follows: 50% and 70% for 30 min each at 4 °C, 90% for 30 min at 23 °C, and 4 changes of 100% for 30 min each at 23 °C. After these dehydration processes, the samples were continuously dehydrated in 100% ethanol at 23 °C over 8 h. Infiltration: the samples were infiltrated with propylene oxide 2 times for 30 min each and were put into 50:50 mixture of propylene oxide and resin (Quetol-651: Nisshin EM Co., Tokyo, Japan) for 4 h, then they were transferred to a 100% resin over 8 h. Embedding and polymerization: the samples were polymerized at 60 °C for 48 h. Ultra-thin sections: the polymerized resins were ultra-thin sectioned at 80 nm with a diamond knife using an ultramicrotome (Ultracut UCT: Leica, Vienna, Austria) and the sections were mounted on copper grids. They were stained with 2% uranyl acetate at 23 °C for 15 min, and then they were washed with distilled water followed by being secondary-stained with Lead strain solution (Sigma-Aldrich) at 23 °C for 3 min. Observation and imaging: the grids were observed by a TEM (JEM-1400Plus: JEOL Ltd., Tokyo, Japan) at an acceleration voltage of 100 kV. Digital images (3296 × 2472 pixels) were taken with a CCD camera (EM-14830RUBY2: JEOL Ltd).

### Evaluation of Young’s modulus and tensile strength

The multilayered cell-plastics were analyzed to evaluate the Young’s modulus and tensile strength using a tensile strength tester (TesTex, Zurich, Switzerland). To prepare for the test, the cell-plastics were cut into rectangles (3 mm × 5 mm) to fit the tensile strength tester. The crosshead rate was 1.00 mm·min^−1^. The values of the Young’s modulus (*E*) of the cell-plastics were calculated using the following formula:

$$E\, = \,\left( {W/A} \right)\,/\,\left( {X/L} \right).$$

where *W*, *A*, *X*, and *L* represent the weight, cross-sectional area, displacement during tensile test, and length of sample, respectively. The values of *W* and *X* were obtained during the tensile strength tests. The values of *A* were calculated as the product of the width (3 mm) and thickness, which were obtained from the SEM observations of the small pieces of samples cut by scissors. *L* was set as 5 mm for both samples. The stress and strain, defined as *W* divided by *A* and *X* divided by *L*, respectively, were calculated. From the tensile strength tests, we plotted the stresses with respect to the strains.

## Results

**Fig. 3:**
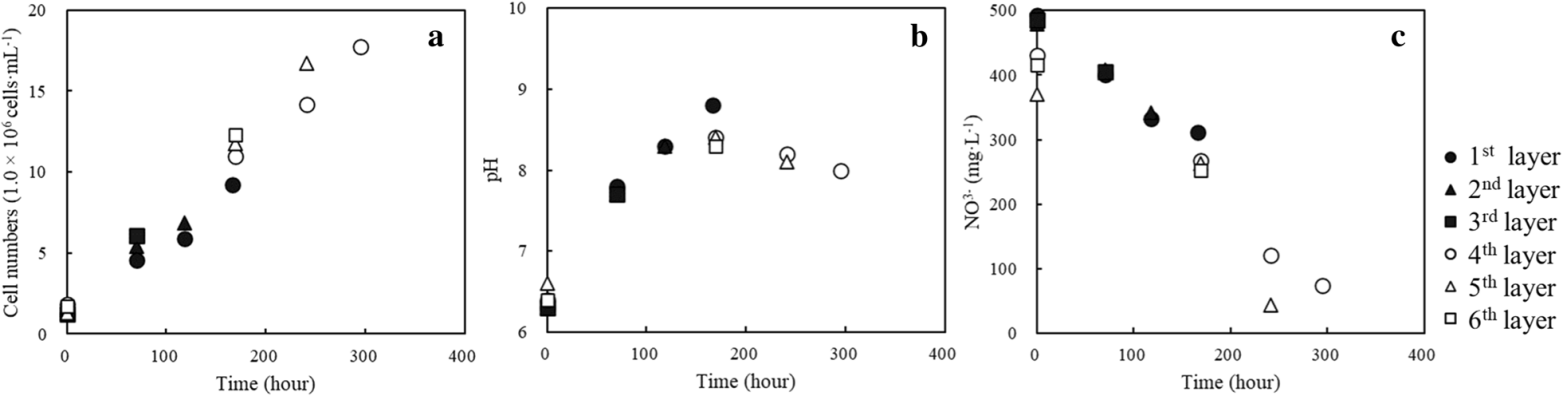
Broth condition of *C. reinhardtii* in each culturing system Time-course profiles of **a** cell number, **b** pH, and **c** nitrate concentration are shown as closed circle (1st layer), closed triangle (2nd layer), closed square (3rd layer), opened circle (4th layer), opened triangle (5th layer) and opened square (6th layer). Harvested points were 70 h (1st layer), 118 h (2nd layer), 166 h (3rd layer), 169 h (4th layer), 241 h (5th layer) and 295 h (6th layer), respectively

*Chlamydomonas reinhardtii* was cultured in MB6N to supply cells as raw materials for the cell-plastics. The cell growth was stable and reached 1.8 × 10^6^ cells·mL^−1^ at 70 h as the 1^st^ sampling point and 1.8 × 10^7^ cells·mL^−1^ at 295 h as the 6th sampling point (Fig. 3a). Although the cultivation of *C. reinhardtii* by Bonente et al. (2012) needed over 240 h (10 days) to reach 1.0 × 10^7^ cells·mL^−1^ under similar conditions to ours, the cultivation in this study attained over 1.0 × 10^7^ cells·mL^−1^ at 169 h, indicating effective cultivation of the strain. The cell supply for the cell ingredients was enough by using only 50–500 mL of broth because the monolayer plastics needed 9.0 × 10^8^ cells. Stable culturing normally shows an increasing pH during the initial growth of *C. reinhardtii* due to the depletion of CO_2_ in the broth (Kosourov et al. 2003). The broth in our study also displayed an increasing pH of 6.4 to 8.8 from the initial culture to 166 h, indicating a stable and activated growth (Fig. 3b). The nitrate concentration decreased reciprocally with increasing cell growth, showing that the cells grew stably (Fig. 3c). Due to nitrogen source depletion in the broth, the cell components of *C. reinhardtii* would be considerably altered (Gargouri et al. 2017). However, in our study, all broths did not experience nitrogen depletion; thus, they were not expected to show drastic changes in the cell components. In fact, the cell components in our study indicated 7.8–11.6% lipids, 35.1–40.5% carbohydrates, and 14.5–21.8% proteins (Table 1), which were similar to those derived from stable cultivation by other researchers (Cakmak et al. 2012; Longworth et al. 2012; Wang et al. 2015). According to these results, the cells harvested as cell ingredients in each culturing condition exhibited similar physical strengths.

**Table 1:** Ratio of cell component for each cell layer

	Lipid	Carbohydrate	Protein
1st cell layer	11.6 ± 3.0	40.5 ± 5.3	19.7 ± 4.7
2nd cell layer	7.8 ± 3.0	40.4 ± 1.2	21.8 ± 7.1
3rd cell layer	9.4 ± 0.2	39.9 ± 0.9	18.9 ± 3.7
4th cell layer	10.1 ± 0.2	38.2 ± 0.8	20.5 ± 7.4
5th cell layer	10.3 ± 0.3	37.0 ± 2.3	14.5 ± 1.5
6th cell layer	9.8 ± 0.4	35.1 ± 2.4	19.5 ± 1.3

Values are the averages of three replicated experiments, ± SD

**Fig. 4:**
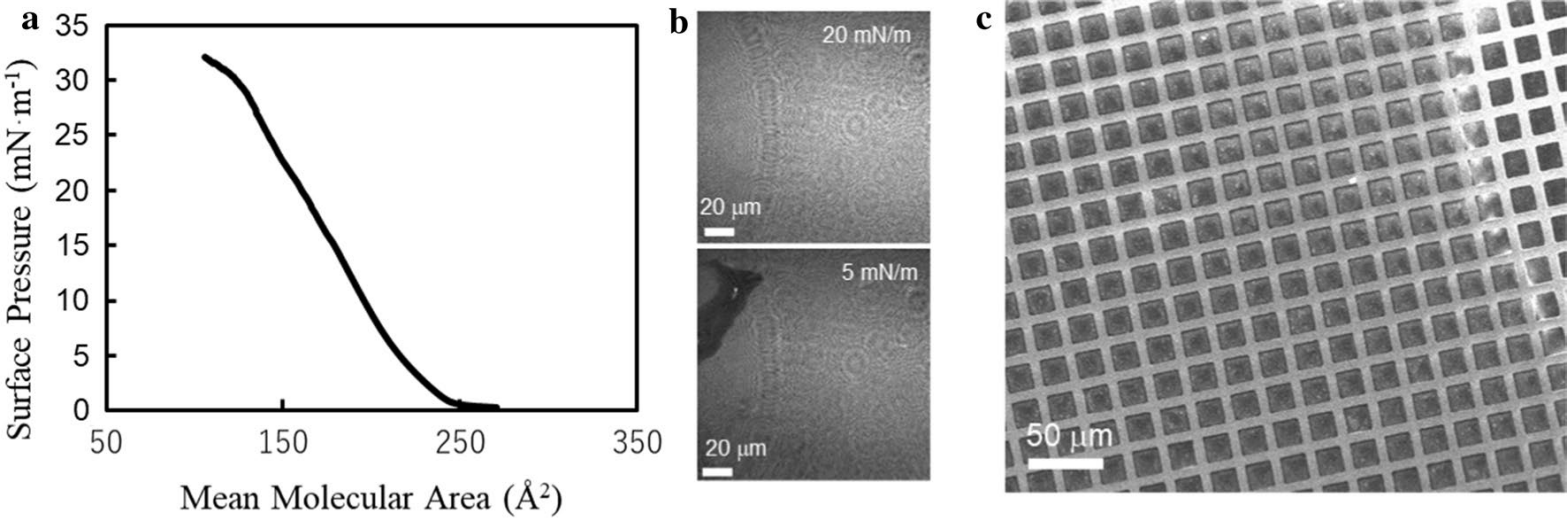
Molecular design and synthesis of 2DP **a** surface pressure-mean molecular area isotherm, **b** Brewster angle microscopy images of the water surface after diffusion of 1 at surface pressure of 5 mN·m^−1^ and 20 mN·m^−1^, and **c** SEM image of UV-irradiated monolayer film on a TEM grid

Before application on the cell layer, we prepared the 2DP at the air/water interface using an LB trough as a pre-experiment. After spreading a chloroform solution of monomer **1** (2.2 × 10^−4^ M) at the air/water interface in the LB trough at room temperature, compression on the water surface was performed at a constant speed of 3 mm·min^−1^. Coincidentally, a surface pressure (SP) versus mean molecular area (MMA) isotherm was recorded (Fig. 4a), and BAM observations on the surface were made (Fig. 4b). In the isotherm, the value of SP increased with an MMA of approximately 250 Å^2^. At an SP of 15 to 20 mN·m^−1^, the MMA reached 160–180 Å^2^, which corresponds to the unit cell area (approximately 170 Å^2^) estimated from the molecular modeling. In addition, based on the BAM images, we found that an incomplete monolayer was formed at an SP of 5 mN·m^−1^ because a nonhomogeneous image was obtained, as shown in the lower part of Fig. 4b. In contrast, at an SP of 20 mN·m^−1^, no defects in the monolayer were observed (upper part of Fig. 4b). Based on these results, we considered that the expected monolayer of monomer **1** was formed. Next, we performed polymerization of the monolayer at the air/water interface by photo-irradiation to connect the adjacent cinnamate units for the formation of a cyclobutane ring (Fig. 2c). After diffusion of monomer **1** at the air/water interface using the method described above, the surface was compressed up to an SP of 20 mN·m^−1^, and that value was maintained. To connect the reactive units, UV light was irradiated at 356 nm (Chung et al. 1991) for 2 h. After irradiation, the photo-irradiated monolayer was transferred onto a TEM grid. Figure 4c depicts the SEM image of the TEM grid with the photo-irradiated monolayer. It was revealed that a large area of the grid was covered with the photo-irradiated monolayer, although some defects were observed (refer to the small area in the right side of Fig. 4c). Therefore, the photo-irradiated monolayer shows sufficient strength to be placed on the grid.

## Discussion

**Fig. 5:**
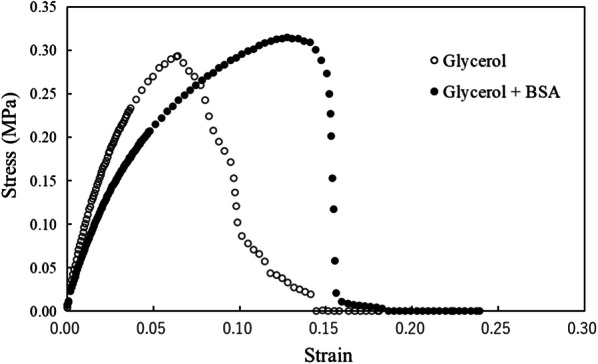
Young’s modulus and tensile strength Stress–Strain curves of cell-plastic (Gly) (white circle) and cell-plastic (Gly-BSA) (Black circle). Their tensile strengths (maximum stresses) and Young’s modulus (slope of the initial growth) are detected to be 0.29 MPa and 9.0 MPa (white circle), and 0.32 MPa and 6.2 MPa (Black circle), respectively

**Fig. 6:**
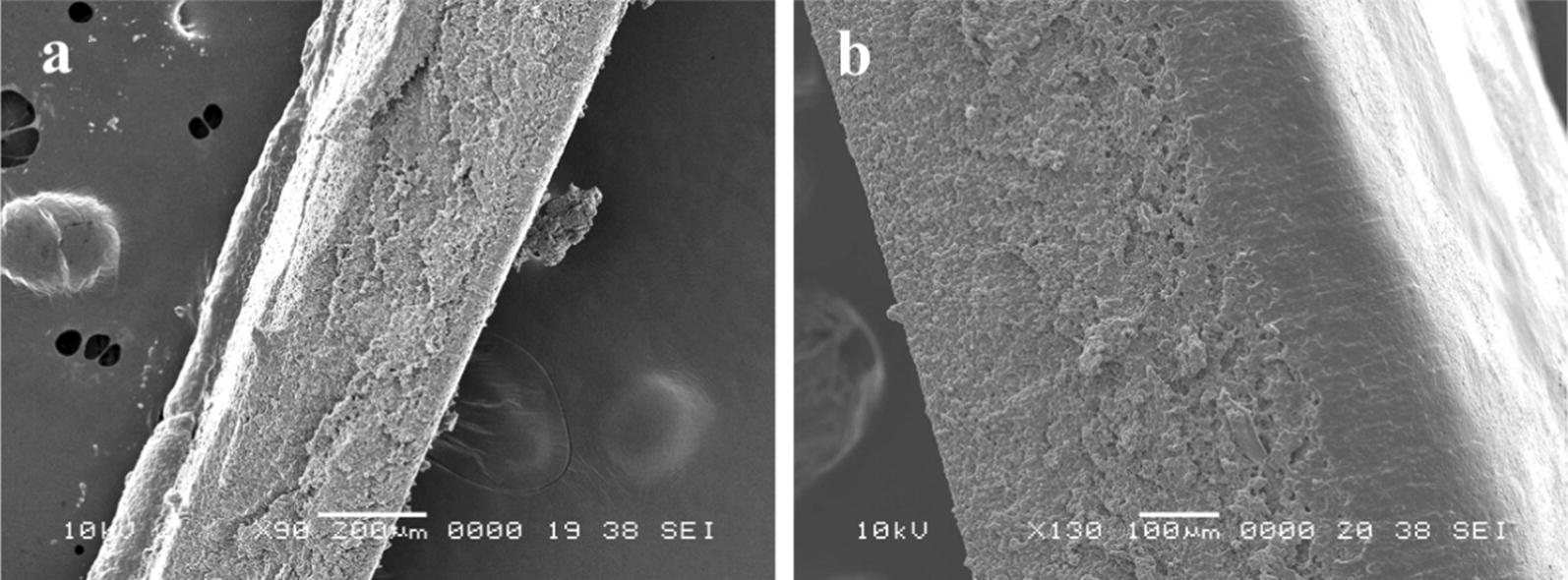
Measuring thickness using SEM image of cross sections Thickness of each cell-plastic is evaluated using SEM image of cross section. **a** Cell-plastic (Gly) and **b** cell-plastic (Gly-BSA) under magnifications of × 90 and × 130, respectively

For preparing the cell-plastics, we used a glass Petri dish (ϕ 5.5 cm) as a template for the fabrication of the monolayer cell-plastics following the method described in “Construction of the monolayer cell-plastic with an intercellular filler” because the method for preparing 2DP using the LB trough caused deformation of the cell layers due to long-term contact with water. To evaluate the potential of the prepared cell-plastics as a substitute to oil-based plastics, we performed tensile strength tests using samples of the cell-plastics, i.e., cell plastics using glycerol (cell-plastic (Gly)) and using a mixture of glycerol and BSA (cell-plastic (Gly-BSA)) as fillers. Figure 5 presents the plots of stresses versus strains, which were calculated from the results of the tensile strength tests. The thicknesses of cell-plastic (Gly) and cell-plastic (Gly-BSA) were found to be 460 μm and 820 μm, respectively, by SEM observations (Fig. 6). Based on the plots, the values of the tensile strength, which denote the maximum stress until breakage of the material, and Young’s modulus, which indicate the rigidity of the material, were obtained. The former and the latter were derived as the maximum value and the slope of the initial growth of the plot, respectively. The tensile strengths of cell-plastic (Gly) and cell-plastic (Gly-BSA) were estimated to be 0.29 MPa and 0.32 MPa, respectively, which are relatively small compared to those of polyethylene (10–30 MPa), polypropylene (35.5 MPa), and polyethylene terephthalate (69 MPa) (Brandrup et al. 1999). In general, polymer materials have high mechanical strengths at the macroscale owing to the interactions between polymer chains, such as van der Waals interactions (Kleis et al. 2005; Kleis et al. 2005). In contrast, for the cell-plastics, the poor mechanical strength was attributed to the weak interactions between their components, although self-supporting films were obtained. In addition, the values of Young’s modulus were calculated to be 9.0 MPa and 6.2 MPa for cell-plastic (Gly) and cell-plastic (Gly-BSA), respectively. These values are relatively small compared to those of polyethylene (200–1400 MPa), polypropylene (1380 MPa), and polyethylene terephthalate (2200 MPa) (Brandrup et al. 1999). These results signify that the fabricated cell-plastics have a higher deformability than commodity plastics.

**Fig. 7:**
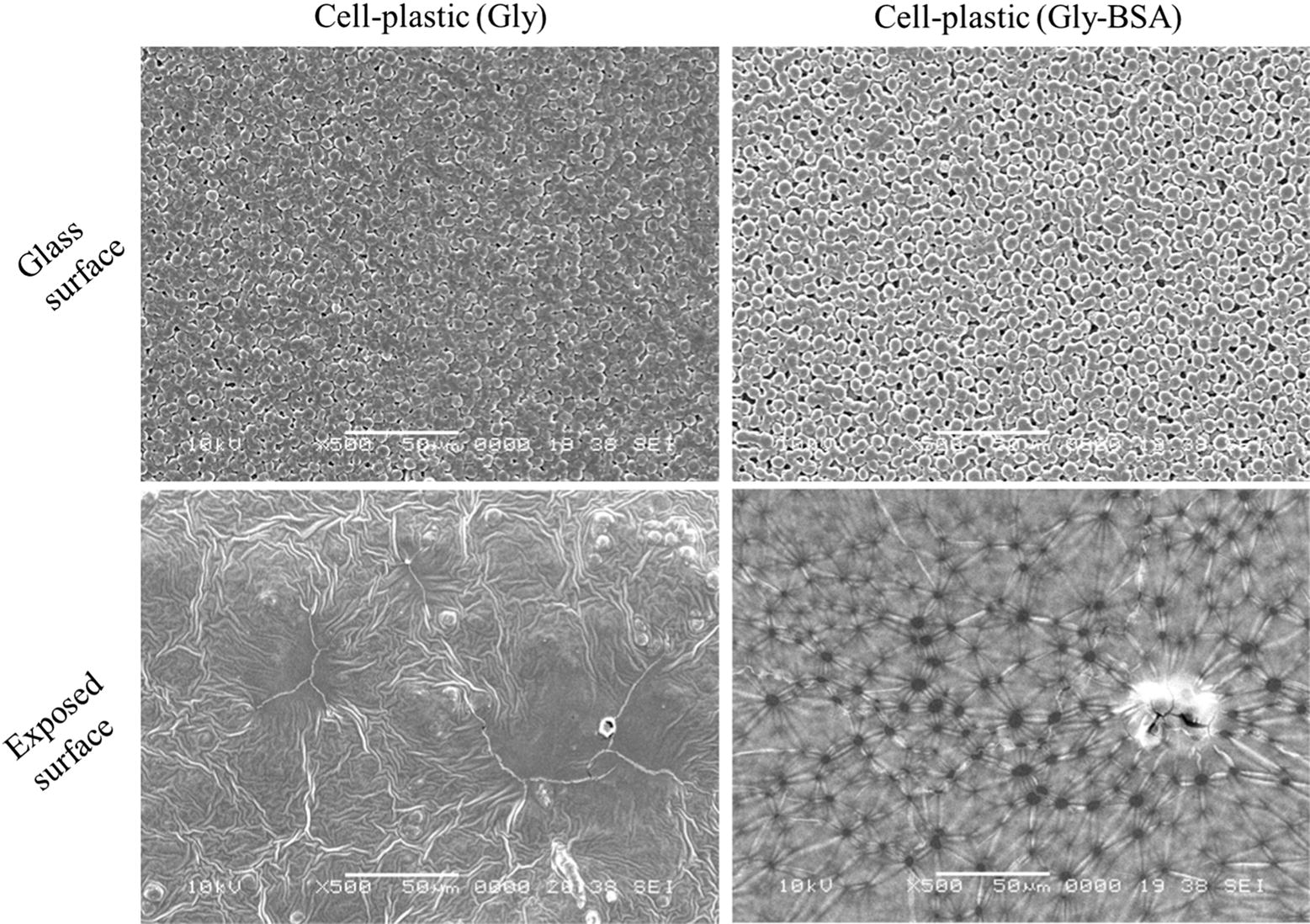
SEM images of surfaces of cell-plastic (Gly) and cell-plastic (Gly-BSA) SEM images of surfaces of cell-plastic (Gly) and cell-plastic (Gly-BSA) on left and right column, respectively. Additionally, SEM images of different surfaces are shown (upper row: glass surface; lower row: exposed surface). All magnifications were × 500

**Fig. 8:**
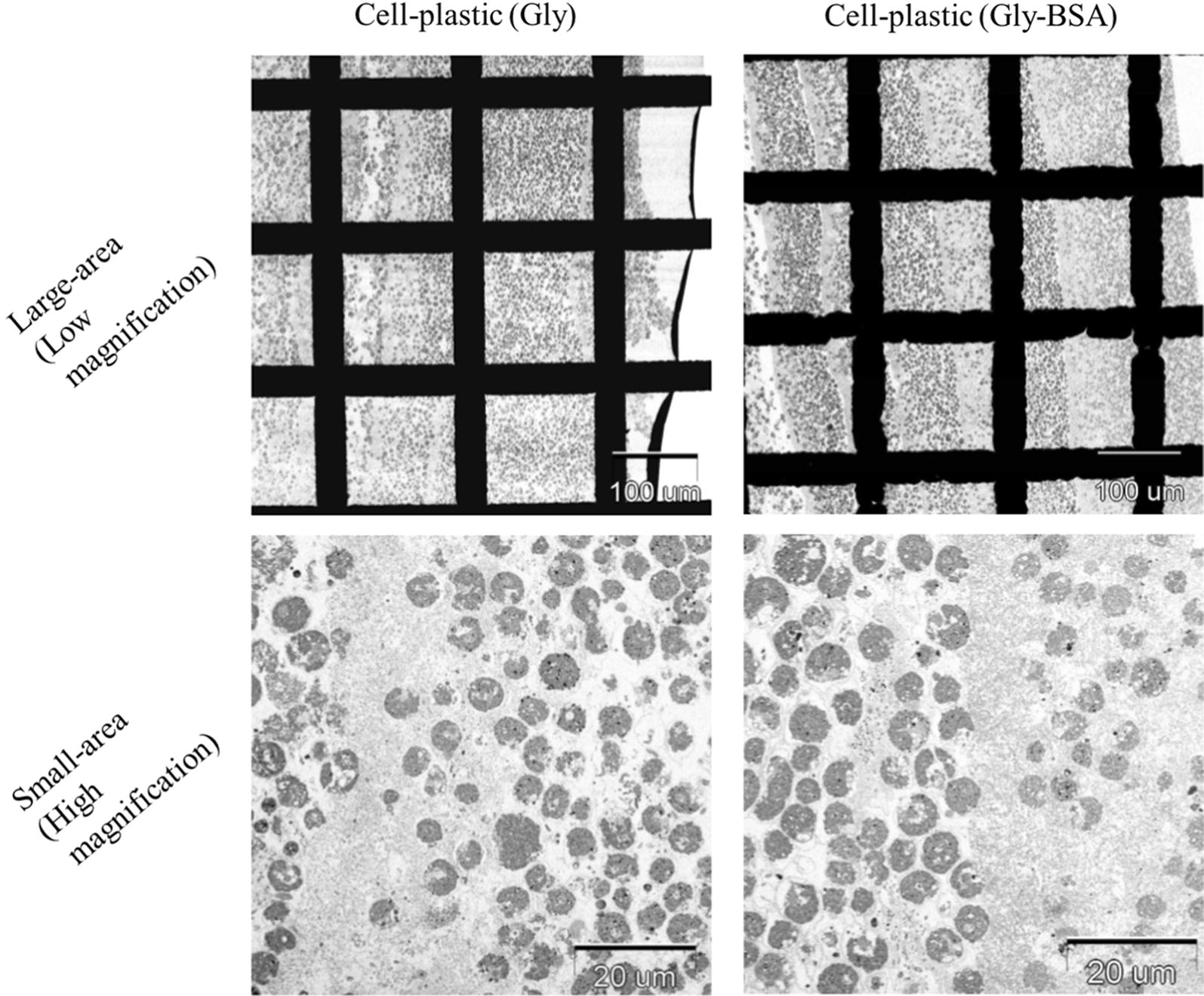
TEM images of cross sections of cell-plastic (Gly) and cell-plastic (Gly-BSA) TEM images of cell-plastic (Gly) and cell-plastic (Gly-BSA) on left and right column, respectively. Additionally, TEM images under different magnifications are shown (upper row: large area (magnification of × 126); lower row: small area (magnification of × 842))

In the comparison between the mechanical strengths of cell-plastic (Gly) and cell-plastic (Gly-BSA), it was found that there was no significant difference between their tensile strengths; however, the Young’s modulus of cell-plastic (Gly) was larger than that of cell-plastic (Gly-BSA). To investigate their structures at the microscale, we examined the SEM and TEM images of the cell-plastic samples, as illustrated in Figs. 7 and 8, respectively. Surprisingly, we found that, from the SEM images of surfaces of both cell-plastics (Fig. 7), the surface that contacted with the glass Petri dish (glass surface) and the other one exposed to the atmosphere (exposed surface) exhibited different structures. In the case of cell-plastic (Gly), a spherical shape of cells was observed for the glass surface (upper left part of Fig. 7), whereas for the exposed surface, a crumpled structure without the spherical-shaped cells was obtained (lower left part of Fig. 7). In contrast, for cell-plastic (Gly-BSA), although the cells maintained their spherical shape on the glass surface (upper right part of Fig. 7), the exposed surface showed a porous structure having 10–20 µm pores arranged at random positions (lower right part of Fig. 7). It is thought that the drying process under vacuum for the fabrication of the cell-plastics had affected the elution of cell contents, causing deformation of the surface structures of the cell-plastics. To support this hypothesis, we examined the SEM images of the cells without a drying process under vacuum. As displayed in Additional file 1: Figure S1, it was found that the cells maintained their spherical shapes. Moreover, in the case of cell-plastic (Gly-BSA), we considered that the porous structure, i.e., a sponge-like structure, would give less rigidity compared to that of cell-plastic (Gly), although unfortunately, we did not completely understand why the porous structure was formed only for cell-plastic (Gly-BSA). On the other hand, Fig. 8 exhibits the TEM images of cross sections of the samples of cell-plastic (Gly) and cell-plastic (Gly-BSA). It was revealed that two types of layers were stacked for both cases. One is a layer filled by the cells that maintained their spherical shapes, which showed *Chlamydomonas* cells according to previous report (Ho et al. 2015). Its thickness was found to be approximately 100 µm. The other is a relatively thin layer with a thickness of approximately 5 µm. Some particulate substances considered to be cell components are observed in the thin layers, which indicate that a mixture of 2DPs and the cell components is formed in the thin layer. We concluded that, although there was no remarkable structural difference between the stacking structures of cell-plastic (Gly) and cell-plastic (Gly-BSA) which affects the Young’s modulus, cell-plastics with a stacking structure were fabricated without slippage between layers.

In summary, a novel cell-plastic was fabricated by utilizing the robustness of the cell wall of *C. reinhardtii*. Tests of the Young’s modulus and tensile strength revealed that the mechanical strength of the cell-plastic was dependent on the intercellular filler, suggesting the need for selecting a suitable filler for a specific application. Additionally, the SEM images showed that the *C. reinhardtii* cells were deposited on the surface of the cell-plastics, and the TEM images proved that the multiple layers in the cell-plastics consisted of a cell layer and 2DP. These results indicate the possibility of producing novel sustainable plastics by using environmentally friendly green algal cells as ingredients. Subsequent research and development of cell-plastics will be expected based on this study.

## Supplementary information

**Additional file 1: Figure S1.** SEM images of surfaces of cell-plastics without intercellular filler. Magnifications were ×1,000. **Figure S2**. Synthesis of monomer **1**. **Figure S3.**^1^H and ^13^C NMR spectra of **3** in CDCl_3_ at 25 °C. **Figure S4.**^1^H and ^13^C NMR spectra of **1** in CDCl_3_ at 25 °C.

## Data Availability

We admit availability of data and material.
