# Supplementary material for: Construction of cell-plastics as neo-plastics consisted of cell-layer provided green alga Chlamydomonas reinhardtii covered by two-dimensional polymer
Source: AMB Express. 2020 Jun 10;10:112. doi: 10.1186/s13568-020-01046-y (PMC7286992; doi:10.1186/s13568-020-01046-y)
Supplement: Supplementary file 1 — Additional file 1: Figure S1. SEM images of surfaces of cell-plastics without intercellular filler. Magnifications were ×1,000. Figure S2. Synthesis of monomer 1. Figure S3.1H and 13C NMR spectra of 3 in CDCl3 at 25 °C. Figure S4.1H and 13C NMR spectra of 1 in CDCl3 at 25 °C. [file 13568_2020_1046_MOESM1_ESM.docx]

**AMB Express**

**The title:**

Construction of cell-plastics as neo-plastics consisted of cell-layer provided green alga *Chlamydomonas reinhardtii* covered by two-dimensional polymer

**The full names of the authors (do not include academic degrees)**

*Akihito Nakanishi*^†1,2^, Kohei Iritani* ^3^, Yuri Sakihama^4^, Nanami Ozawa^2^, Ayano Mochizuki^2^, Marina Watanabe^2^*

*(*Co-first author, ^†^corresponding author)*

**The author's institutional affiliations where the work was conducted**

*^1^Graduate School of Bionics, Tokyo University of Technology, Hachioji, Japan*

*^2^School of Bioscience and Biotechnology, Tokyo University of Technology, Hachioji, Japan*

*^3^Department of Applied Chemistry, School of Engineering, Tokyo University of Technology, Hachioji, Japan*

*^4^Tokyo University of Technology, Hachioji, Japan*

Address correspondence to:

*A. Nakanishi*

*Graduate School of Bionics, Tokyo University of Technology,*

*School of Bioscience and Biotechnology, Tokyo University of Technology*

*1404-1 Katakuramachi*

*Hachioji, Tokyo, 192-0982, Japan*

*Phone: +81 42-637-2427*

*E-mail:* [nakanishiah@stf.teu.ac.jp](mailto:nakanishiah@stf.teu.ac.jp)

**Supplemental data**


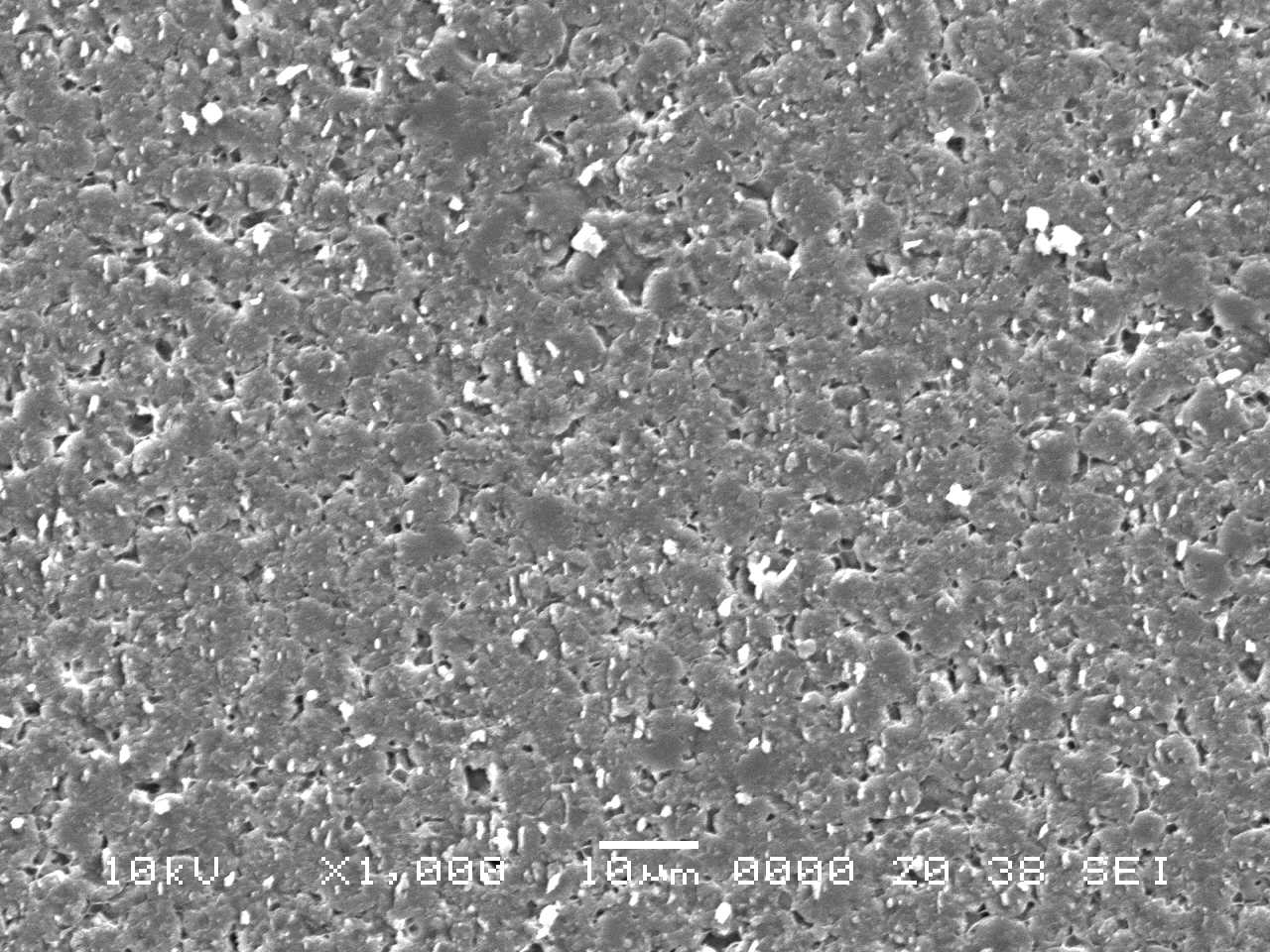


Fig. S1

SEM images of surfaces of cell-plastics without intercellular filler. Magnifications were ×1,000.

**Experimental Details for Synthesis of compounds**

**1. General**

All solvents were distilled before use. All commercially available reagents were used as received. Fig. S2 shows synthetic scheme of monomer **1**. Compound **4** was prepared according to previous reports (Hosseini et al. 2016). ^1^H (400 MHz) and ^13^C (100 MHz) NMR spectra were measured on a Bruker UltraShield Plus 400 spectrometer. When chloroform-*d* were used as a solvent, the spectra were referenced to residual solvent proton signals in the ^1^H-NMR spectra (7.26 ppm) and to the solvent carbon signals in the ^13^C-NMR spectra (77.0 ppm). Figs. S3 and S4 show ^1^H and ^13^C NMR spectra of compound **3** and monomer **1**, respectively. Preparative HPLC separation was undertaken with a JAI LC-9204 recycling chromatographs using 600 mm × 40 mm JAIGEL-1H-40 and 2H-40 (for JAI LC-9204) GPC columns with CHCl_3_ as the eluent.

**2. Synthesis of monomer 1**

Fig. S2 Synthesis of monomer **1**.

**Syntheses of Compounds**

**Compound 3**

A solution of vanilline (2.50 g, 16.4 mmol) and **2** (1.16 g, 1.82 mmol) in DMF (30 mL) was heated under 100 °C in the presence of K_2_CO_3_ (2.24 g, 16.2 mmol) for 24 h. After cooling to room temperature, the mixture was added to water dropwise for reprecipitation. After the filtration, compound **3** (1.86 g, 96%) was given as a light yellow solid.

^1^H-NMR (400 MHz, CDCl_3_, 25 °C) δ 9.83 (s, 6H), 7.38 (d, *J* = 1.8 Hz, 6H), 7.34 (dd, *J* = 8.1 Hz and 1.8 Hz, 6H), 7.03 (d, *J* = 8.2 Hz, 6H), 5.36 (s, 12H), 3.80 (s, 18H); ^13^C-NMR (100 MHz, CDCl_3_, 25 °C) δ 190.7, 153.0, 150.2, 137.7, 131.0, 126.3, 112.9, 109.2, 65.0, 55.7.

**Monomer 1**

Compound **3** (249 mg, 0.234 mmol), **4** (752 mg, 2.76 mmol) were stirred in cyclohexane (2.5 mL). To the solution, pyridine (0.175 mL, 2.17 mmol) and aniline (0.250 mL, 2.74 mmol) were added. After string at 80 °C for 18 h, the mixture was added to water. The product was extracted by CH_2_Cl_2_. The organic phase was washed with water and brine, and dried over Na_2_SO_4_. After removal of the solvent under vacuum, the crude mixture was purified by preparative HPLC and reprecipitation (CH_2_Cl_2_/hexanes) to give **1** (268 mg, 49%) as an orange solid.

^1^H-NMR (400 MHz, CDCl_3_, 25 °C) δ 7.58 (d, *J* = 16 Hz, 6H), 7.09–6.80 (m, 18H), 6.29 (d, *J* = 16 Hz, 6H), 5.30 (brs, 12H), 4.26–4.06 (m, 12H), 3.71 (brs, 18H), 1.91–0.95 (m, 120H), 0.92–0.79 (m, 18H); ^13^C-NMR (100 MHz, CDCl_3_, 25 °C) δ 167.1, 150.1, 144.2, 137.9, 129.1, 122.1, 116.5, 114.6, 113.6, 110.1, 65.3, 64.7, 55.5, 31.9, 29.61, 29.58, 29.5, 29.32, 29.29, 28.7, 26.0, 22.7, 14.1.

**3. ^1^H and ^13^C Spectra of Compounds**

**
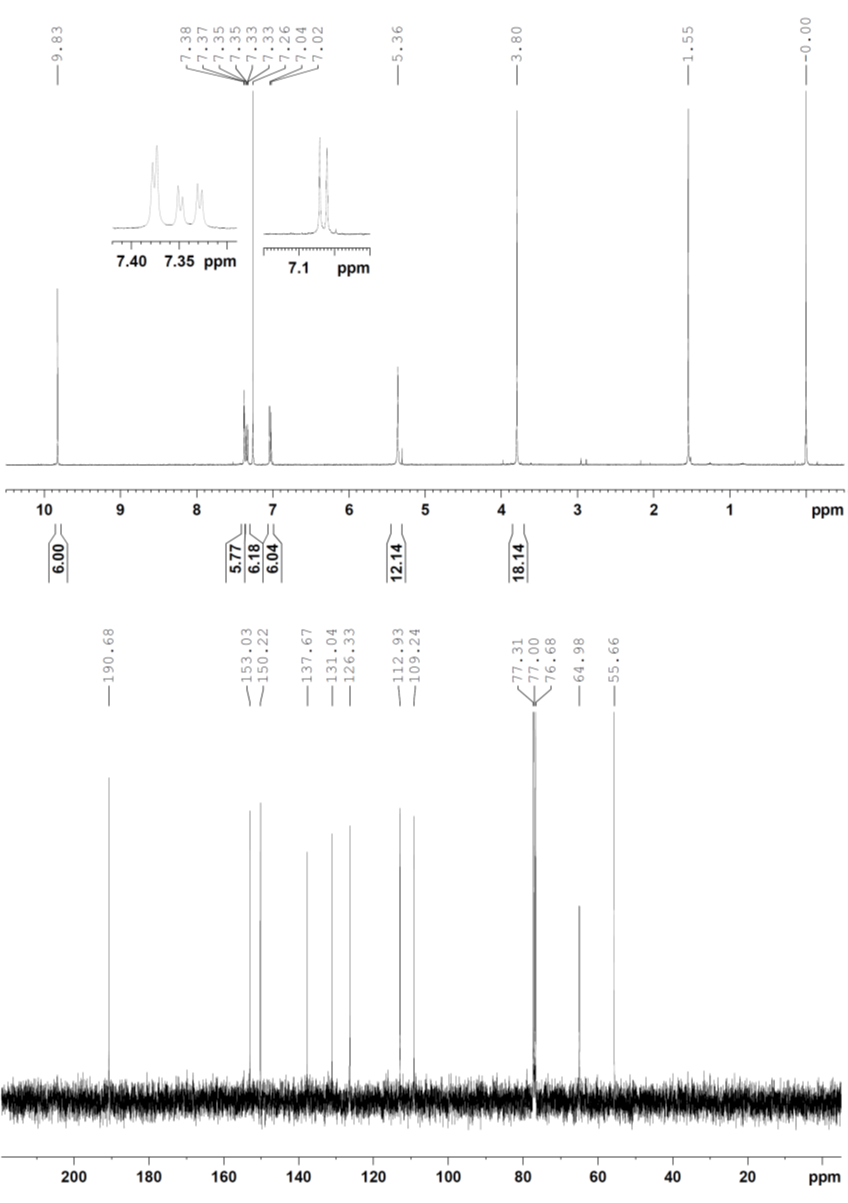
**

Fig. S3 ^1^H and ^13^C NMR spectra of **3** in CDCl_3_ at 25 °C.


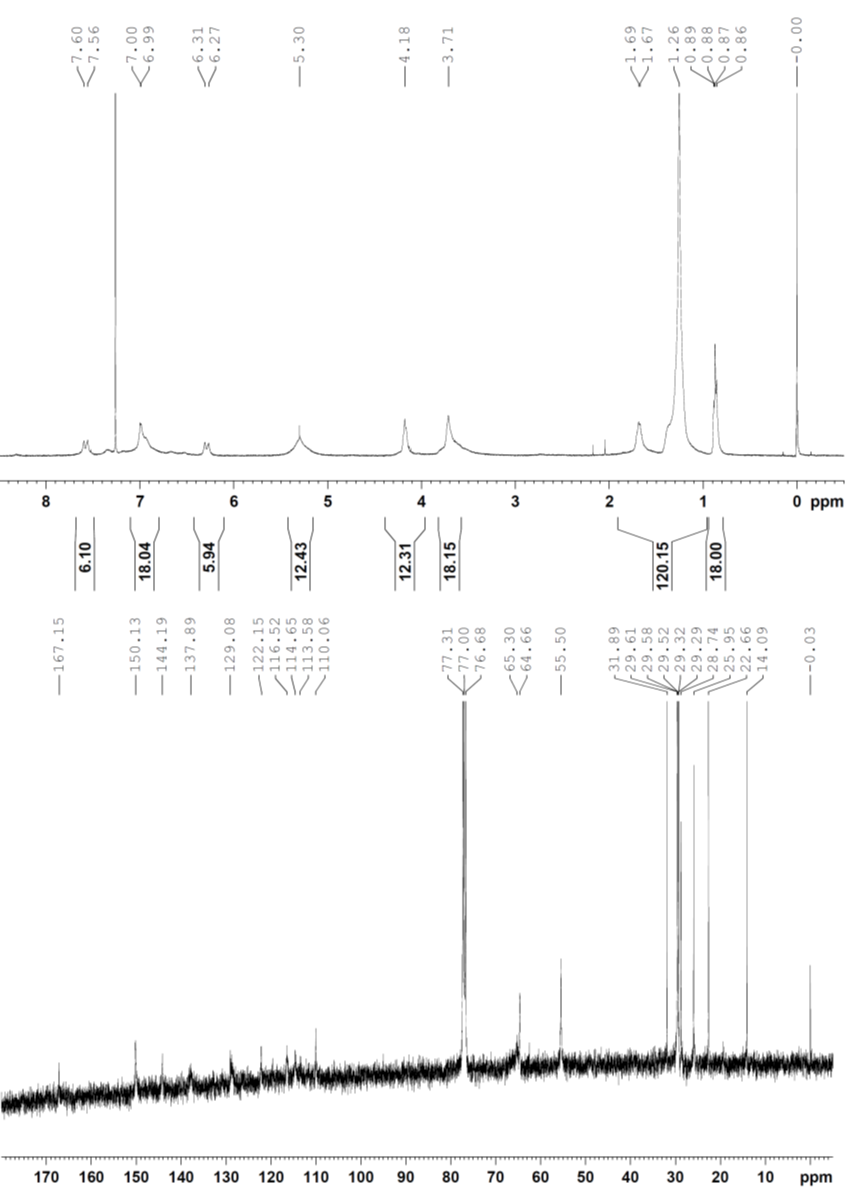


Fig. S4 ^1^H and ^13^C NMR spectra of **1** in CDCl_3_ at 25 °C.

**Reference**

Hosseini R, Moosavi F, Rajaian H, Silva T, Magalhães e Sikva D, Soares P, Saso L, Edraki N, Miri R, Borges F, Firuzi O (2016) Discovery of neurotrophic agents based on hydroxycinnamic acid scaffold. Chem Biol Drug Des 88:926-937
